# Supplementary material for: Stage-dependent cerebrocerebellar communication during sensorimotor processing
Source: Nat Commun. 2025 Oct 3;16:8812. doi: 10.1038/s41467-025-64592-8 (PMC12494962; doi:10.1038/s41467-025-64592-8)
Supplement: Supplementary file 1 — Supplementary Information [file 41467_2025_64592_MOESM1_ESM.pdf]

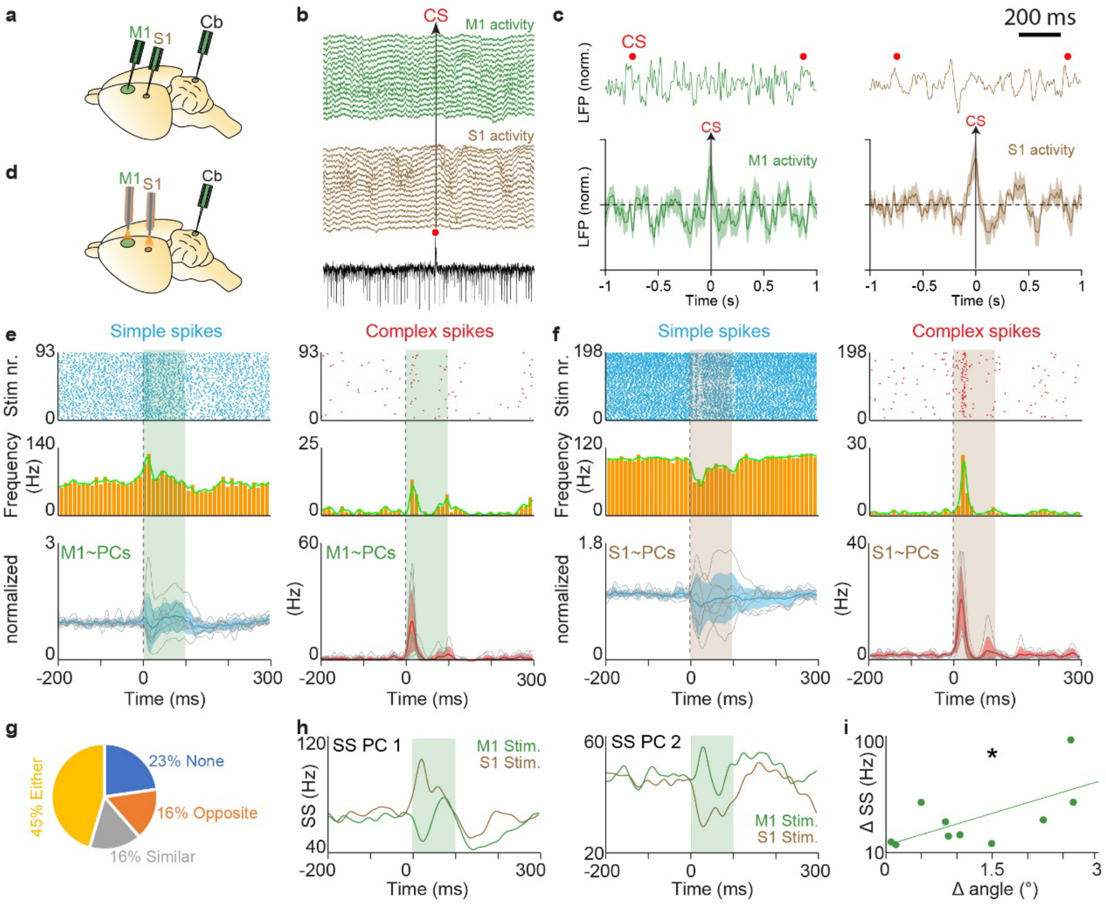

a
M1
S1
Cb
b
CS
M1 activity
S1 activity
c
200 ms
CS
LFP (norm.)
M1 activity
S1 activity
Time (s)
d
e
Simple spikes
Complex spikes
Stim nr.
Frequency (Hz)
normalized
(Hz)
M1~PCs
Time (ms)
f
S1~PCs
g
23% None
16% Opposite
16% Similar
45% Either
h
SS PC 1
SS PC 2
SS (Hz)
M1 Stim.
S1 Stim.
Time (ms)
i
Δ SS (Hz)
Δ angle (°)

Figure S1

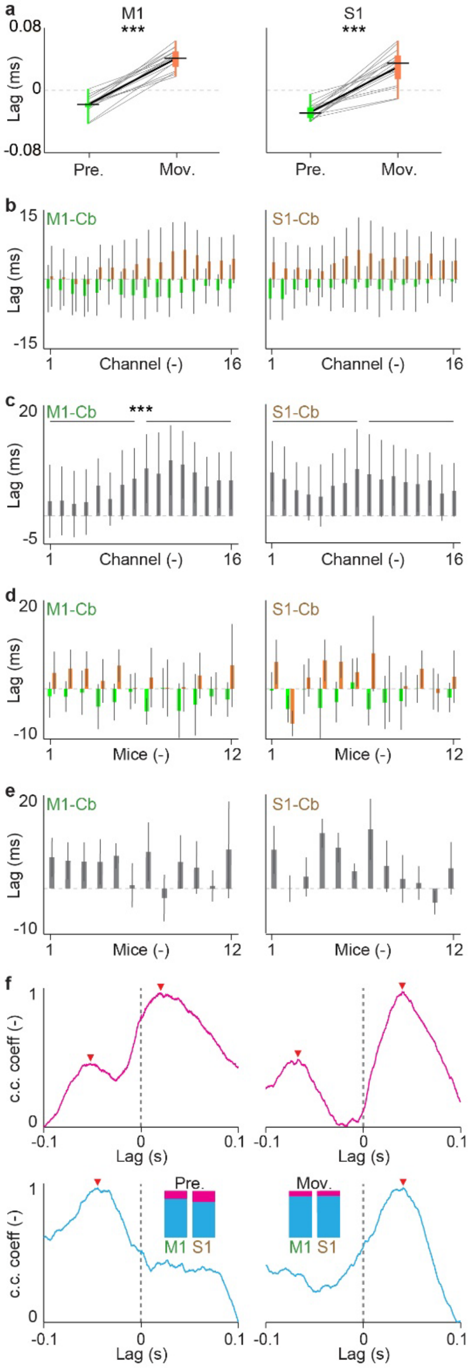
a
M1
S1
***
0.08
0
-0.08
Lag (ms)
Pre.
Mov.
b
15
-15
M1-Cb
S1-Cb
Channel (-)
1
16
c
20
-5
d
20
-10
Mice (-)
12
e
f
c.c. coeff (-)
Lag (s)
-0.1
0.1
Pre.
Mov.
M1
S1

Figure S2

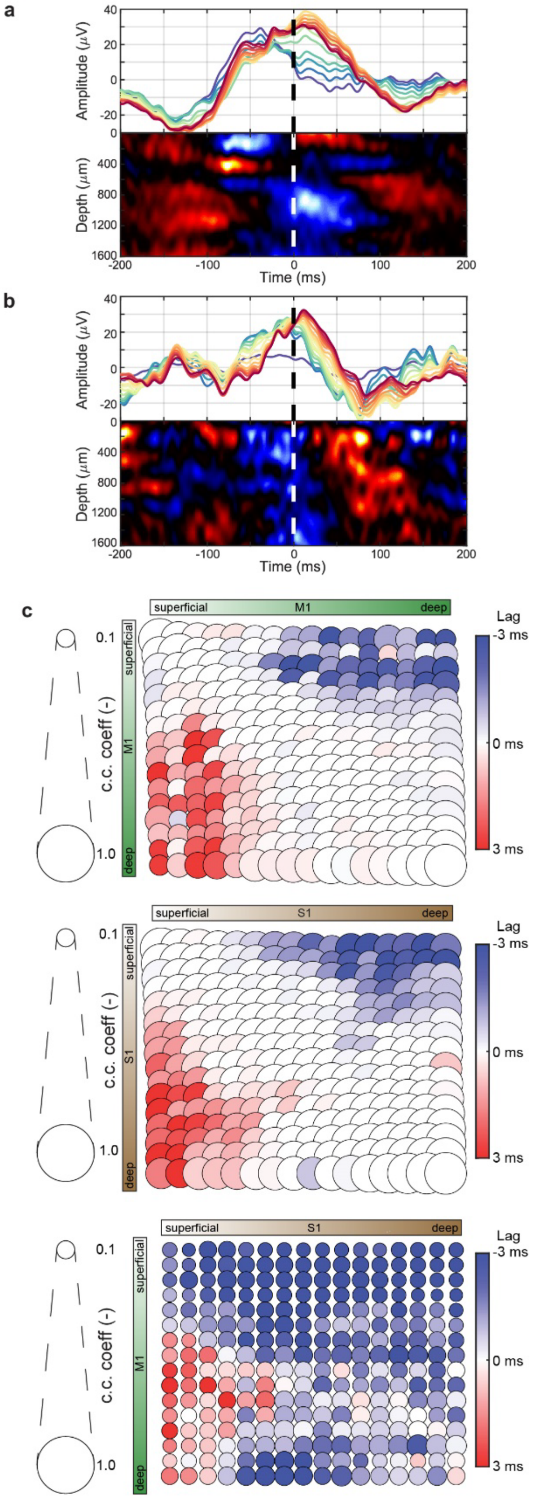
a
Amplitude (μV)
Depth (μm)
Time (ms)
b
Amplitude (μV)
Depth (μm)
Time (ms)
c
superficial
M1
deep
S1
c.c. coeff (-)
0.1
1.0
Lag
-3 ms
0 ms
3 ms

Figure S3

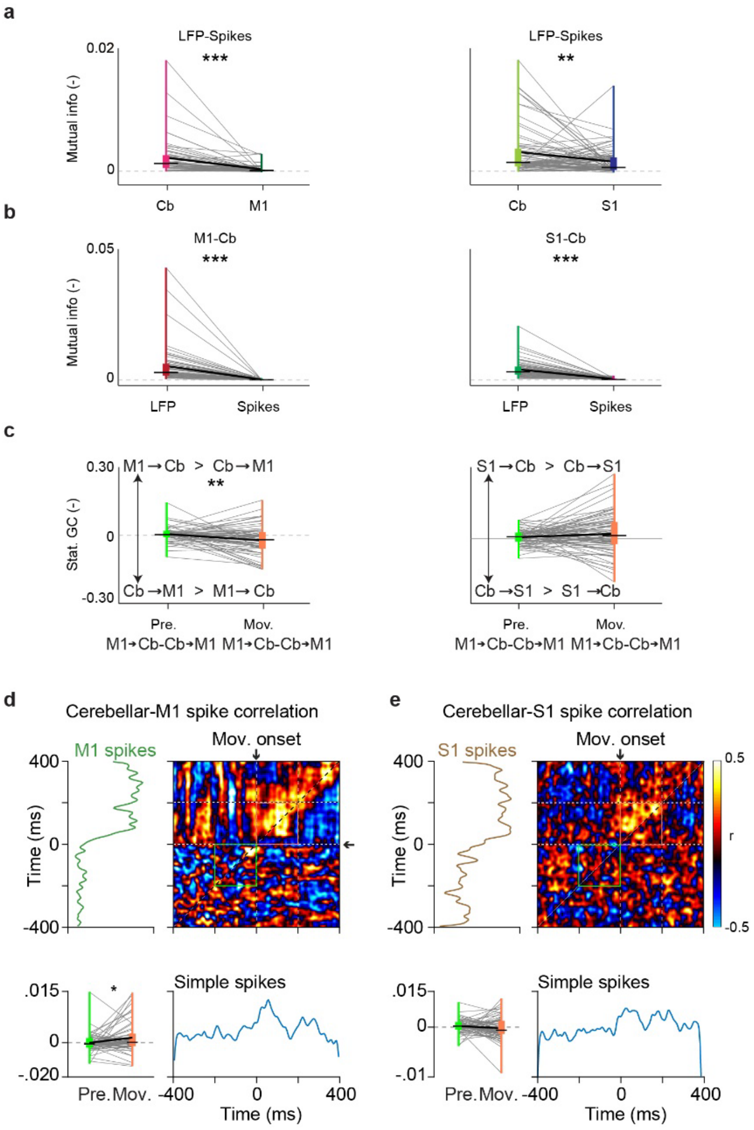
a
LFP-Spikes
***
LFP-Spikes
**
Mutual info (-)
0.02
0
Cb
M1
Cb
S1
b
M1-Cb
***
S1-Cb
***
Mutual info (-)
0.05
0
LFP
Spikes
LFP
Spikes
c
0.30
M1→Cb > Cb→M1
**
Stat. GC (-)
0
-0.30
Cb→M1 > M1→Cb
S1→Cb > Cb→S1
Cb→S1 > S1→Cb
Pre.
Mov.
M1→Cb-Cb→M1 M1→Cb-Cb→M1
Pre.
Mov.
M1→Cb-Cb→M1 M1→Cb-Cb→M1
d
Cerebellar-M1 spike correlation
M1 spikes
Mov. onset
400
Time (ms)
0
-400
e
Cerebellar-S1 spike correlation
S1 spikes
Mov. onset
400
Time (ms)
0
-400
0.5
r
-0.5
.015
*
0
-.020
Pre.
Mov.
Simple spikes
-400
0
400
Time (ms)
.015
0
-.01
Pre.
Mov.
Simple spikes
-400
0
400
Time (ms)

Figure S4

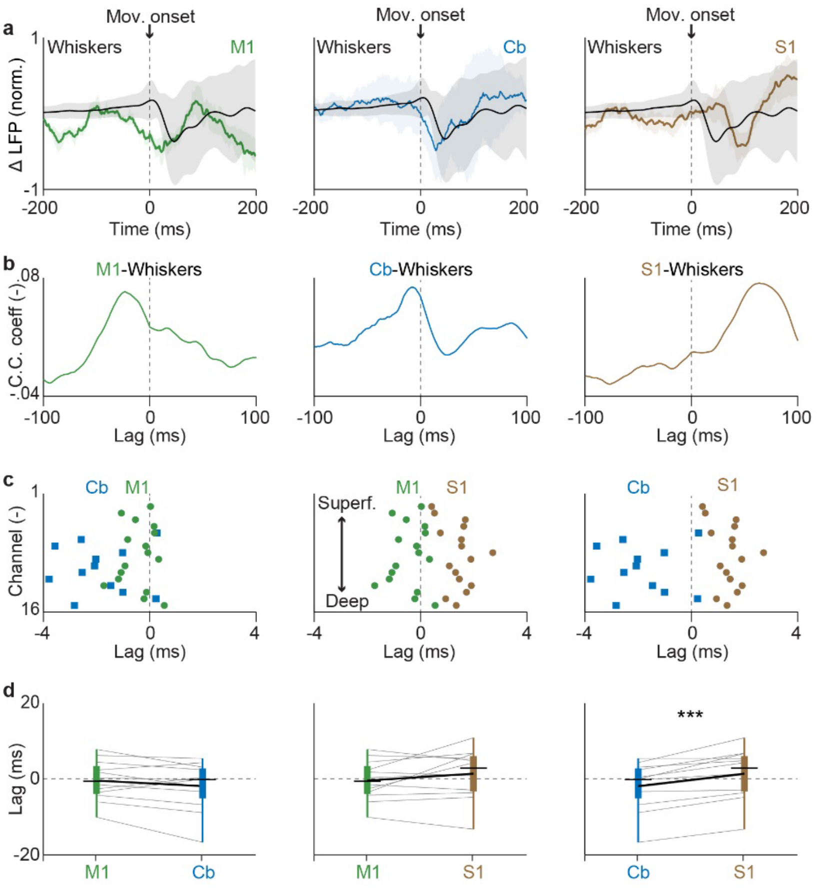
a
Mov. onset
Whiskers
M1
Cb
S1
Δ LFP (norm.)
Time (ms)
b
M1-Whiskers
Cb-Whiskers
S1-Whiskers
C.C. coeff (-)
Lag (ms)
c
Channel (-)
Superf.
Deep
d
Lag (ms)
***

Figure S5

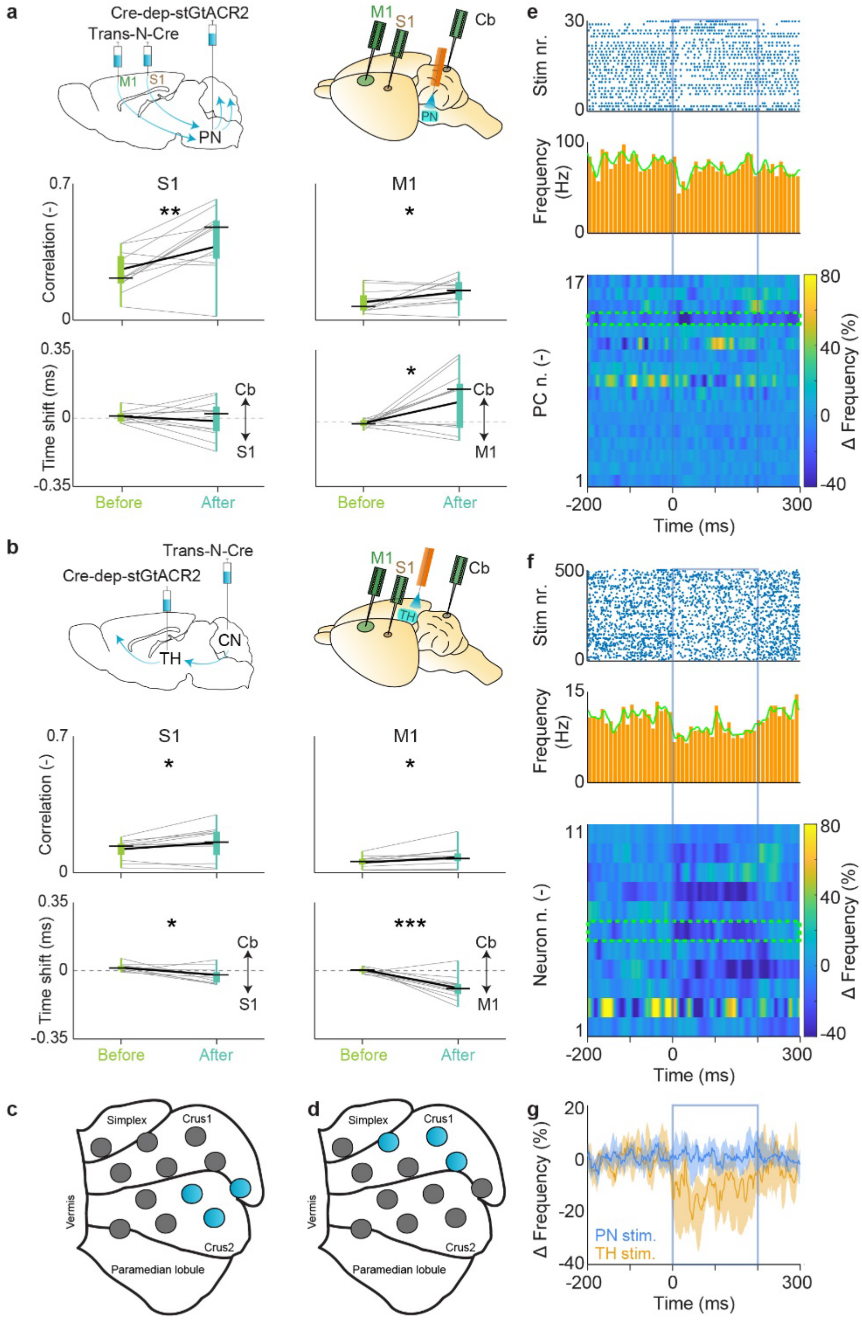
a
Cre-dep-stGtACR2
Trans-N-Cre
M1
S1
PN
M1
S1
Cb
PN
S1
M1
Correlation (-)
0.7
0
**
*
Time shift (ms)
0.35
0
-0.35
*
Cb
S1
Cb
M1
Before
After
Before
After
b
Trans-N-Cre
Cre-dep-stGtACR2
CN
TH
M1
S1
TH
Cb
S1
M1
*
*
*
***
Cb
S1
Cb
M1
Before
After
Before
After
c
Simplex
Crus1
Vermis
Crus2
Paramedian lobule
d
Simplex
Crus1
Vermis
Crus2
Paramedian lobule
e
Stim nr.
30
0
Frequency (Hz)
100
0
PC n. (-)
17
1
80
40
0
-40
Δ Frequency (%)
-200
0
300
Time (ms)
f
Stim nr.
500
0
Frequency (Hz)
15
0
Neuron n. (-)
11
1
80
40
0
-40
Δ Frequency (%)
-200
0
300
Time (ms)
g
Δ Frequency (%)
20
0
-20
-40
PN stim.
TH stim.
-200
0
300
Time (ms)

Figure S6

Figure S1. Integration of cortico-pontine and cortico-olivary sensorimotor pathways in the lateral cerebellum

(a) Schematic of the experimental approach of M1, S1 and cerebellar Purkinje cell recording. **(b)** An example of local field potential (LFP) signal recorded throughout M1 (green) and S1 (brown) while simultaneously recording PC activity. **(c)** Top: examples of LFP signal from one M1 and one S1 channel and two CSs (indicated by the red dots). Bottom: LFP in M1 and S1 averaged around complex spike time (n=10 PCs). **(d)** Schematic of the experimental approach with M1 and S1 stimulation while recording cerebellar Purkinje cell activity. **(e)** Top left: raster plot showing the simple spikes around 100 ms M1 stimulation, highlighted in green. Middle left: Peri-stimulus time histogram (PSTH) from the raster plot. Bottom: Average simple spike modulation for all PCs responsive (see method) to M1 stimulation (10 out of 44). Each gray line represents an individual PC and the blue line is the average with the shaded area as plus and minus 1 standard deviation. On the right side is the same but for complex spikes responses. **(f)** Similar to e, but for the 8 PCs responsive to S1 stimulation (indicated with the brown shaded area). **(g)** Pie chart depicting the percentage of PC that significantly modulate their SS upon S1 and M1 stimulation. Some PC were sensitive to "Either" M1 or S1 and some to "None" of them. Other PCs were sensitive to both M1 and S1, but their response could be "Similar" in the directionality of the SS modulation (i.e., facilitation-facilitation or suppression-suppression) or "Opposite" (i.e., facilitation-suppression or suppression-facilitation). **(h)** Two examples of PC "Opposite" modulation for S1 and M1 are shown. **(i)** Scatter plots of the max values of simple spike and whisker modulation induced by M1 stimulation ($p = 0.044$, Pearson correlation).

Figure S2. The flip of the phase of cerebrocerebellar communication throughout the cortical layers and cerebellar locations

(a) The mean phase relations of cerebellar and cerebral activity of all 12 mice as in Figure 1f, but grouped per cortical channel for both the Pre-movement (green) and Movement (orange) stage (M1-cb $p < 0.0001$, S1-cb $p < 0.0001$; paired TTEST). Each line represents the mean phase relation at a specific depth of M1 (left) and S1 (right), with channel 1 and channel 16 representing the most superficial and deepest channel, respectively. **(b)** The means obtained at each depth for both stages are also shown with the relative variability (error bars representing the standard deviations, SDs). **(c)** Difference between the phase relation during the Movement stage minus that of the Pre-movement stage, with comparison between the 8 shallower channels versus the 8 deeper channels. The change of the phase relation was bigger for the activity recorded in the deeper channels, compared to that of the superficial channels, for M1 and not for S1 (M1-cb $p = 0.0004$, S1-cb $p = 0.7725$; paired TTEST). **(d)** The mean and the SDs of the phase relation is shown per mouse during the Pre-movement (green) and the Movement (orange) stage. The levels of variability are shown across the 12 different cerebellar locations and across mice (each pair of bars reflects a mouse). **(e)** The means and SDs of the

differences of the phase relations (Movement stage minus Pre-movement stage) per mouse. **(f)** Different examples of normalized cross-correlograms with either two continuous (top left) or separated (top right) peaks or just one single peak (bottom). The bar plot shows the ratio between the two types. The number of peaks of the mean cross-correlogram was quantified per cerebellar location in each mouse (see also Methods). During the Pre-movement stage double peaks were present in 17.0% M1-cb and 23.7% S1-cb cross-correlograms. During the Movement stage, double peaks were present in 10.7% M1-cb and 11.4% S1-cb cross-correlograms. Therefore, the large majority of the cross-correlograms had only a single peak.

Figure S3. Validation of the calculation of the phase relation across the local field potential signals of different channels

(a) At the top, the average M1 LPF around the movement onset is shown for all 16 channels, from superficial (blue) to deep (red) channels for an exemplary mouse. Below, is the current source density (CSD), showing that, especially during the Movement phase, the current flows from superficial to deep channels. **(b)** Similar to panel a, but for S1 LFP signals showing also the current flowing towards the deeper channels. **(c)** Cross-correlation between the 16 channels within M1 (top) during the Movement stage. The size of each circle represents the peaks of the cross-correlograms for each pair of channels averaged for 12 mice. The color of the circle represents the average phase relation of the peak of the cross-correlation; the cross-correlation is computed relative to the vertical axis, meaning that blue indicates that the channel on the vertical line precedes that on the horizontal line, and that conversely, the red indicates that the channel on the vertical line follows that on the horizontal line. The cross-correlation between the 16 channels within S1 is shown in the middle. Note that in both M1 and S1 activity of the superficial channels preceded that of the deep channels. The bottom panel is the same, but for the cross-correlations between all channels of M1 and S1. Here the cross-correlation is computed relative to M1, implying that negative values in blue indicate that the phase relation is such that M1 correlated better with delayed S1, so changes in M1 LFP signals precede changes in the S1 signals. The plots indicate that the activity of the superficial layers of M1 precedes that of S1 during the Movement stage.

Figure S4. Validation of the local field potential results with spikes from single units

(a) Mutual information between the cerebellar local field potential (LFP) and the simple spikes of PCs compared to that between the cerebral LFP and pyramidal spike activity. The LFP-spike mutual information was higher in the cerebellum than that in the cerebral cortex (Cb-M1 $p < 0.00001$, Cb-S1 $p = 0.00144$; Mann-Whitney U-test). **(b)** Mutual information between the LFPs of the cerebellum and cerebral cortex was higher than the mutual information between spike activity (Cb-M1 $p < 0.00001$, Cb-S1 $p < 0.00001$; Mann-Whitney U-test). **(c)** Difference between the statistic values of the Granger causality test from M1 to the cerebellum minus

that from the cerebellum to M1 (left) in the Pre-movement stage compared to those of the Movement stage. This analysis is based on the spike activity and each line represents a pair of PC and M1 neurons simultaneously recorded. During the Pre-movement stage, the Granger causality was higher from M1 to the cerebellum and during the Movement stage higher from the cerebellum to M1 (p = 0.00509; paired TTEST). On the right, the same but for S1 and cerebellar spike activity (p = 0.16857; paired TTEST). **(d)** Correlation matrix analysis [23,24,32] showing a positive correlation of simple spike firing (blue trace at the bottom shows convolved peri-stimulus time histogram triggered on movement onset) and spike activity of a presumptive pyramidal neuron recorded in M1 (green trace) on a trial-by-trial analysis (see Methods for details). The correlation coefficient (r) over the dashed 45° indicates the correlation between PCs and M1 neuronal spiking without time shift. The red-yellow blob above the diagonal indicates a positive correlation such that in trials (i.e., movement onsets) with more PC spikes at time 1, there were more spikes in the M1 neuron at time 2. Note that this occurs mostly after movement onset. Before movement onset, the red blob is mostly below the diagonal (indicating that more spikes in the M1 neuron at time 1 correlated with more PC simple spikes at time 2). We quantified all the r values above the diagonal within the Pre-movement stage (top-left half of the green square) and subtracted those below the diagonal (bottom-right half of the green square). Similarly, we subtracted the r values above and below the diagonal within the Movement stage (orange square). The resulting differences are plotted in the left bottom corner for comparison (each line represents one pair of neurons). In line with the results on LFP data (Figure 1f) and the Granger causality analysis of the spikes (panel c), the phase relation during the Pre-movement stage was different from that during the Movement-stage (p = 0.0232; paired TTEST). **(e)** Similar to panel d, but for the correlation between the PC simple spikes and spike activity of neurons from S1. Here, no significant difference could be detected between the Pre-movement and Movement stages (p = 0.1090, paired TTEST).

Figure S5. Different phase relations of activity in motor cortex, cerebellum and sensory cortex with respect to the whisker movements

(a) The mean LFP signals of motor cortex (M1, green on the left), cerebellum (Cb, blue in the middle), and sensory cortex (S1, brown on the right) are shown together with the mean whisker position for one exemplary mouse. The shaded areas indicate the standard deviations. **(b)** Examples of cross-correlograms between M1 LFP (left), Cb LFP (middle) and S1 LFP (right) and whisker movement calculated in the epoch of maximal movement (i.e., in the 20-170 ms period after movement onset; see also Methods). **(c)** The phase relations, i.e., lead or lag of the cross-correlogram peaks, are shown for each of the 16 M1, 12 Cb, and 16 S1 recording channels. Each square (Cb) or circle (M1 and S1) represents the average phase relation across all 12 mice. To show the difference across the different cerebral cortical layers, the y values are sorted according to the M1 and S1 channel depth, with the superficial and deeper

channels being presented at the top and bottom parts of each panel, respectively. The Cb values follow the same reference frame imposed by the cerebral cortical distribution of the channels. **(d)** Comparing the phase relations between M1, Cb, S1 LFPs and whisker movements at a population level (each line represents a mouse). Whisker movement had a similar phase relation with M1 LFPs and Cb LFPs ($p = 0.1329$; paired TTEST, left) as well as with M1 LFPs and S1 LFPs ($p = 0.1827$; paired TTEST, middle). Conversely, the whisker movement had a more negative phase relation with the Cb LFPs than with the S1 LFPs ($p = 0.0002$; paired TTEST, right), indicating that the cerebellar and S1 LFPs on average precede and follow the movement, respectively.

Figure S6. Stimulation of pons or thalamus alters cerebrocerebellar dynamics

(a) Schematic of the viral transfection and experimental approach (top). Transneuronal-Cre was injected into M1 and S1 and cre-dependent-stGtACR2 into the pons (PN). During the experiment, the cerebellum (Cb), M1 and S1 are recorded while manipulating PN activity. Lower panels show the correlation strength between S1 and Cb (left) and that between M1 and Cb around stimulation onset (Before = -200 to 0 and After = 0 to 200 relative to stimulus onset), for one exemplary mouse during the resting period (see Methods). Each line represents the cross-correlogram's maximum values for each cerebellar recording location (S1 $p = 0.0019$, M1 $p = 0.0322$; paired TTEST). The phase relation of the correlation between the neocortex and Cb does not significantly change for S1, but it does change for M1 (S1 $p = 0.6705$; M1 $p = 0.0263$; paired TTEST). A positive phase relation means that the strongest correlation occurs between a cerebellar signal and a delayed neocortical signal, while a negative phase relation shows the strongest correlation between a neocortical signal and a delayed cerebellar signal. **(b)** Similar to panel a, but for thalamic stimulation. A schematic of the viral transfection and experimental approach is shown (top). Transneuronal-Cre is injected into the cerebellar nuclei (CN) and cre-dependent-stGtACR2 into the thalamus (TH). The activity of the cerebellum and that of M1 and S1 are recorded while the thalamus is stimulated. Below is the correlation strength between S1 and Cb (left) and between M1 and Cb (right) around TH stimulation onset (Before = -200 to 0 and After = 0 to 200 relative to stimulus onset), for one exemplary mouse during the resting period. Each line represents the cross-correlogram's maximum values for each cerebellar recording location (S1 $p = 0.0297$, M1 $p = 0.0184$; paired TTEST). The average phase relations between neocortex and Cb before and during thalamus stimulation are plotted at the bottom (S1 $p = 0.0143$, M1 $p = 0.0006$; paired TTEST). Note that, especially for the Cb-M1 phase relation, the changes induced by manipulating the ascending pathway via the thalamus are in the opposite direction of those induced by manipulating the descending pathway via the PN. **(c)** Schematic representing which of the cerebellar recording locations matched the model's prediction for the outcomes of the PN stimulation. The recording spots used for the analyses of Figure 4f are highlighted in cyan. **(d)** Similar to c, but for the TH stimulation and the analyses of Figure 5f. **(e)** Raster plot

of the simple spike activity of a Purkinje cell around the moment of PN stimulation (top). Below in the middle panel, we show the relative Peri-stimulus time histogram (PSTH) of the same data with a green line to visualize the changes in firing rate over time. At the bottom, the changes in firing rate are shown as a color map for all 17 Purkinje cells recorded during PN stimulation. The green dashed square indicates the exemplary Purkinje cell shown above. **(f)** Similar to panel e, but for a cerebral cortical neuron recorded during TH stimulation. **(g)** Averages and standard deviations of changes in firing rate of all the neurons during PN stimulation (blue) and TH stimulation (orange).
